# Ultrafast Exciton Dynamics in the Atomically Thin van der Waals Magnet CrSBr

**DOI:** 10.1021/acs.nanolett.3c05010

**Published:** 2024-03-20

**Authors:** Christian Meineke, Jakob Schlosser, Martin Zizlsperger, Marlene Liebich, Niloufar Nilforoushan, Kseniia Mosina, Sophia Terres, Alexey Chernikov, Zdenek Sofer, Markus A. Huber, Matthias Florian, Mackillo Kira, Florian Dirnberger, Rupert Huber

**Affiliations:** † Department of Physics and Regensburg Center for Ultrafast Nanoscopy (RUN), University of Regensburg, 93040 Regensburg, Germany; ‡ Department of Inorganic Chemistry, University of Chemistry and Technology Prague, 166 28 Prague 6, Czech Republic; § Institute of Applied Physics and Würzburg-Dresden Cluster of Excellence, Dresden University of Technology, 01187 Dresden, Germany; ∥ Department of Electrical Engineering and Computer Science, University of Michigan, Ann Arbor, Michigan 48109, United States

**Keywords:** atomically thin solids, van der Waals magnets, anisotropic excitons, ultrafast dynamics, femtosecond near-field microscopy, terahertz

## Abstract

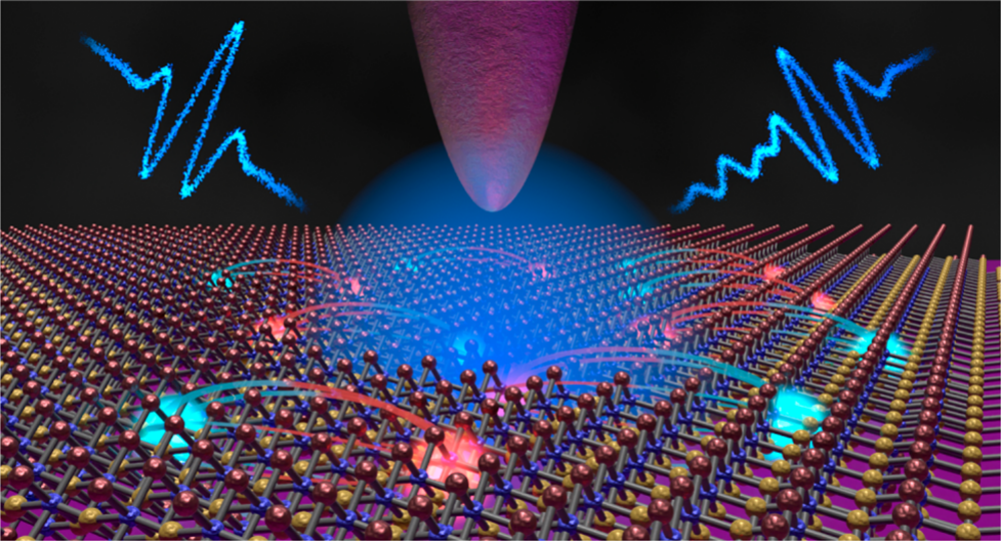

Among atomically thin semiconductors, CrSBr stands out as both its bulk and monolayer forms host tightly bound, quasi-one-dimensional excitons in a magnetic environment. Despite its pivotal importance for solid-state research, the exciton lifetime has remained unknown. While terahertz polarization probing can directly trace all excitons, independently of interband selection rules, the corresponding large far-field foci substantially exceed the lateral sample dimensions. Here, we combine terahertz polarization spectroscopy with near-field microscopy to reveal a femtosecond decay of paramagnetic excitons in a monolayer of CrSBr, which is 30 times shorter than the bulk lifetime. We unveil low-energy fingerprints of bound and unbound electron–hole pairs in bulk CrSBr and extract the nonequilibrium dielectric function of the monolayer in a model-free manner. Our results demonstrate the first direct access to the ultrafast dielectric response of quasi-one-dimensional excitons in CrSBr, potentially advancing the development of quantum devices based on ultrathin van der Waals magnets.

The advent of magnetic two-dimensional crystals hosting strongly bound excitons has initiated an unparalleled development in the realm of quantum materials.^1−6^ In these systems, magnetic order fundamentally influences excitonic properties, such as effective mass, eigenenergies, and polarization, spawning a variety of emergent quantum phenomena.^7−10^ The van der Waals layered magnet CrSBr stands out particularly for its intricate interplay between magnetic order and quasi-one-dimensional electronic and lattice structure,^11−22^ establishing CrSBr as a unique platform for fundamental research and future quantum devices. In CrSBr, electron–hole pairs form highly anisotropic excitons whose real-space wave function extends along the in-plane crystallographic *b* axis. Below its Néel temperature of *T*_N_ = 132 K, bulk CrSBr features in-plane ferromagnetic order among layers, which are antiferromagnetically coupled along the stacking direction, while quasi-one-dimensional excitons are strongly localized within individual layers. The breakdown of magnetic order in the paramagnetic phase, above *T*_N_, should result in a more 2D character of excitons tunneling between adjacent layers. In the monolayer limit, the out-of-plane confinement causes the quasi-one-dimensional nature of excitons to persist even beyond room temperature.

The combination of tight spatial confinement of excitons in the Cr–S chains and the separation of the van der Waals layers by the Br atoms has been reported to result in high exciton binding energies of ∼0.7 and ∼0.1 eV in monolayers and bulk, respectively.^22^ These robust excitons pave the way for applications in optoelectronics and quantum information processing at room temperature. While clear photoluminescence signatures of exciton recombination were identified at a photon energy of 1.37 eV,^22^ the actual size of the single-particle bandgap in CrSBr is still under debate.^23^ Scanning tunneling spectroscopy (STS) measurements have estimated the bulk bandgap to amount to 1.5 ± 0.2 eV,^7,22^ whereas the bandgap of monolayer CrSBr has only been predicted by theory. To selectively prepare and study unbound electron–hole pairs above the bandgap or excitons, pump–probe measurements with tunable excitation wavelengths are hence desirable.

Recent studies of CrSBr have primarily focused on controlling exciton-coupled magnons at radio frequencies using magnetic fields and cavity photons.^9−11^ Meanwhile, the ultrafast dynamics of nonequilibrium electron–hole pairs has remained unexplored, to the best of our knowledge. Particularly, the potential impact of short-lived magnetic fluctuations, so-called paramagnons,^24,25^ on exciton dynamics has not been considered yet. Terahertz (THz) time-domain spectroscopy^26^ provides a unique tool to trace the ultrafast dielectric response of photoexcited electron–hole pairs. However, as the micron-scale lateral dimensions of typical exfoliated CrSBr monolayers are much smaller than the diffraction limit of THz pulses, subwavelength spatial resolution is a prerequisite for conclusive studies.

Here, we employ ultrafast polarization nanoscopy,^27,28^ a technique based on time-resolved near-field microscopy^29−33^ at THz frequencies,^34−37^ to explore the dynamics of electron–hole pairs in paramagnetic CrSBr. Following optical excitation by tunable femtosecond laser pulses, THz probe fields directly trace the dynamics of continuum states and excitons regardless of interband selection rules. Our nanoscopic subcycle approach allows us to resolve the femtosecond decay and the dielectric response of unbound electron–hole pairs and quasi-one-dimensional excitons in bulk and atomically thin CrSBr, for the first time. Furthermore, we observe ultrafast relaxation of excitons in bulk CrSBr in which scattering with paramagnons could occur.

Figure 1a shows an optical micrograph of a typical CrSBr sample exfoliated on a SiO_2_ layer (thickness, 285 nm) fabricated on a p^++^-doped silicon substrate. The depicted area contains both bulk (yellow/blue) and monolayer (ML, faint blue) flakes, which, owing to the structural anisotropy of CrSBr, extend along the crystallographic *a* axis. The micrometer-sized lateral dimensions of the monolayers are much smaller than the diffraction limit of THz pulses. To overcome this mismatch, we couple phase-locked THz waveforms (blue) to the apex of a metallic tip of an atomic force microscope (Figure 1b and section 1 in the Supporting Information). The confined evanescent near field interacts with the sample in a nanoscopic area on the order of the tip’s radius of curvature. Therefore, the nanoscale dielectric response of the sample is imprinted in the scattered THz electric field, which we retrieve by electro-optic sampling (EOS) and demodulation of the signal at the oscillation frequency of the tip.

**Figure 1:**
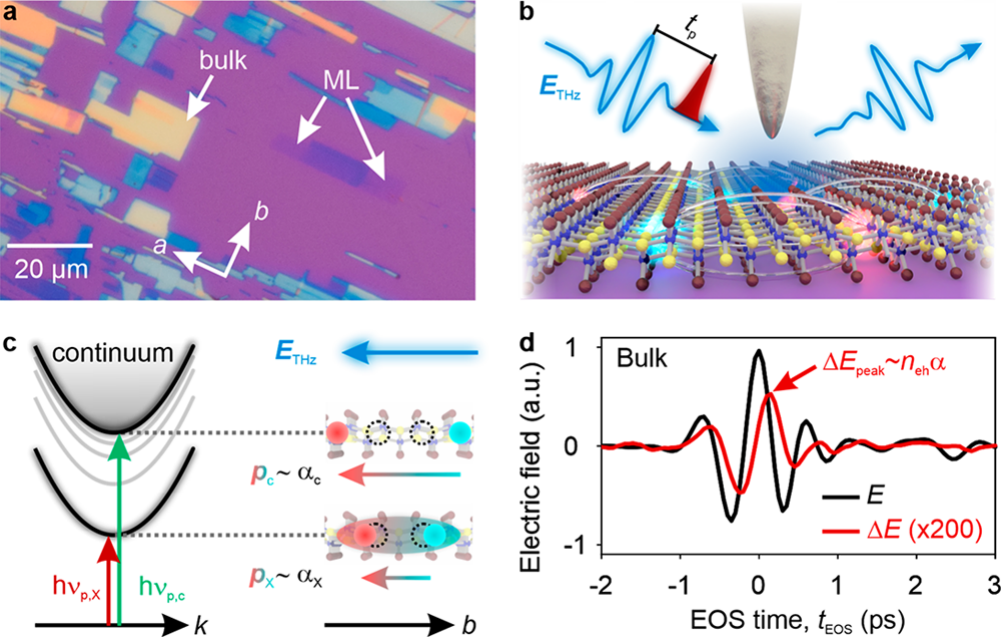
Probing ultrafast electron–hole pair dynamics in bulk and monolayer CrSBr by THz polarization nanoscopy. (a) Optical micrograph of a typical CrSBr sample including bulk (yellow, blue) and monolayer (ML, faint blue) flakes. The crystallographic *a-* and *b*-axes are indicated. (b) Schematic of the THz near-field spectroscopy technique. Optical pump pulses (red) tunable in photon energy generate electron–hole pairs in CrSBr. After a variable delay time, *t*_p_, a phase-locked THz probe transient, *E*_THz_ (blue, left), is coupled into the evanescent near field of a metallic tip. By phase-resolved detection of the scattered THz waveform (blue, right) information about the nanoscale dielectric function of the sample is obtained. (c) Polarization nanoscopy. Tunable pump pulses excite either excitons (red, *h*ν_p,X_) or continuum states (green, *h*ν_p,c_). The THz electric field (blue arrow) polarizes the electron–hole pairs with polarizability α_X_ and α_c_, respectively. (d) Electro-optically detected steady-state scattered THz waveform, *E* (black line), and pump-induced change, Δ*E* (red line), of a bulk CrSBr flake at a pump delay, *t*_p_ = 0.5 ps, as a function of the EOS time, *t*_EOS_.

Moreover, the sample can be photoexcited with ultrashort optical pulses (pulse duration <100 fs) from a noncollinear optical parametric amplifier, allowing us to tune the pump photon energy, *h*ν_p_, between 1.30 and 2.41 eV (section 2 in the Supporting Information). This enables us to selectively inject electron–hole pairs in excitonic or continuum states (Figure 1c, left). The THz electric near field features components along the highly polarizable *b*-axis (blue arrow),^27^ inducing each electron–hole pair to carry a dipole moment, *p*, which is proportional to the polarizability, α. By analyzing the pump-induced change in the scattered THz field, Δ*E*, we can discriminate between highly polarizable unbound electron–hole pairs and excitons, whose polarizability is reduced by Coulomb binding (Figure 1c, right). Importantly, owing to the small pump spot size (∼5 μm), signal contributions from regions far away from the tip are excluded from Δ*E*. Figure 1d displays the electro-optically detected steady-state near-field response of a CrSBr bulk flake, *E* (black curve), together with a typical pump-induced change (red curve), recorded at a pump delay time *t*_p_ = 0.5 ps after photoexcitation with *h*ν_p_ = 1.39 eV. Δ*E* roughly traces *E* with a phase shift of about π/3. The spectra of *E* and Δ*E* will be discussed later in the text.

To gain first insights into the lifetime of electron–hole pairs in bulk and monolayer CrSBr, we record the maximum pump-induced change of the scattered THz transient, which is proportional to the density, *n*_eh_, and polarizability, α, of photoexcited electron–hole pairs, Δ*E*_peak_ ∝ *n*_eh_α, as a function of *t*_p_. We set the pump polarization along the *b*-axis because the corresponding optical interband transitions are dipole-allowed in this direction, while they are forbidden along the *a*-axis.^14^Figure 2a shows the pump–probe dynamics of the THz near-field response (electro-optic delay time, *t*_EOS_ = 0.15 ps) in bulk CrSBr (thickness, 400 nm) for a pump fluence Φ_p_ = 5 mJ cm^–2^ and photon energies 1.30 eV ≤ *h*ν_p_ ≤ 1.81 eV, spanning both the 1s exciton resonance at 1.37 eV and the reported bandgap of 1.5 ± 0.2 eV. To gauge the contribution per electron–hole pair, we divide Δ*E*_peak_ by the respective electron–hole pair density, *n*_eh_, weighted with the finite probing depth of the THz near field (section 3 in the Supporting Information). Irrespective of *h*ν_p_, the pump-induced signal grows abruptly upon photoexcitation, reaches its maximum at *t*_p_ = 0.5 ps, and subsequently decays with biexponential behavior.

**Figure 2:**
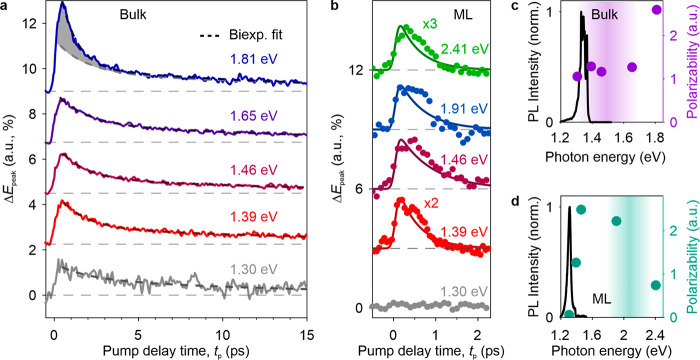
Femtosecond electron–hole pair dynamics in bulk and monolayer CrSBr. (a) Maximal pump-induced change of the near-field response at *t*_EOS_ = 0.15 ps, Δ*E*_peak_, of bulk CrSBr as a function of pump delay time, *t*_p_, for various excitation photon energies (solid lines). The decay of Δ*E*_peak_ is fitted with a biexponential function (dashed lines). The gray shaded region in the top panel shows the excess of the pump-induced signal at early delays with respect to lower excitation photon energies. The data are offset for clarity. (b) Analogous to (a) for the monolayer (ML) limit. Solid lines are calculated with a rate equation model. (c, d) Polarizability of the photoexcited electron–hole pairs as a function of the pump photon energy for bulk (purple circles) and monolayer (teal circles). For context, the low-temperature photoluminescence spectra (PL, solid lines) as well as bandgaps measured with STS (bulk, purple ribbon)^7^ and predicted by GW calculations (ML, teal ribbon)^22^ are shown.

While we observe a slow decay time (1/e), τ_slow_ = 15 ± 3 ps common to all data sets, the fast initial decay strongly depends on *h*ν_p_. For *h*ν_p_ = 1.39, 1.46, and 1.65 eV, we extract an initial decay time, τ_fast_ = 1.6 ± 0.3 ps. However, at *h*ν_p_ = 1.81 eV, this decay takes place faster within τ_fast_ = 1.0 ± 0.1 ps. Moreover, the amplitude of Δ*E*_peak_ is significantly higher (gray area) than that for lower *h*ν_p_, suggesting that different species of electron–hole pairs are involved throughout the decay. For excitation below the bandgap, we expect to initially prepare hot excitons, which relax into the 1s ground state within about 1.6 ps. The thermalization may result from scattering with phonons or short-range spin correlations, that is, paramagnons.^24,25^ We attribute the slow decay common to all photon energies to the recombination of 1s excitons with lifetimes of ∼15 ps. In contrast, the fast decay of the polarization signal at *h*ν_p_ = 1.81 eV is indicative of the formation of 1s excitons; when excitons from energetically more distant bands or unbound electron–hole pairs bind into 1s states, their polarizability is quenched by Coulomb attraction. We will investigate the different excitation scenarios in more detail with complementary measurements presented further below.

Proceeding from bulk to monolayer CrSBr, we estimate that depending on the excitation energy only a few tens to hundreds of electron–hole pairs are probed in the near-field volume of the tip. The excellent sensitivity of our setup enables us to still detect their femtosecond dynamics. We trace Δ*E*_peak_ as a function of *t*_p_ for *h*ν_p_ = 1.30 eV (below the 1s state), 1.39, 1.46, 1.91 eV (above the 1s state), and 2.41 eV (above the calculated bandgap), as shown in Figure 2b. The applied pump fluences were restricted to Φ_p_ ≤ 2.5 mJ cm^–2^, which is safely below the damage threshold of the monolayer. At *h*ν_p_ = 1.30 eV, below the exciton resonance, no pump-induced change in the THz signal is detectable. In contrast, when the pump photon energy is sufficient to excite excitons, we see an abrupt increase of Δ*E*_peak_ followed by an ultrafast, subpicosecond decay. The observed dynamics can thus be assigned to the excitation and decay of excitons in monolayer CrSBr.

As Δ*E*_peak_ changes on time scales comparable to the duration of our pump and gate pulses (∼100 fs), for a quantitative investigation, we simulate the time evolution of Δ*E*_peak_ with a rate equation model comprising a source term given by the pump pulse and a subsequent exponential decay (section 4 in the Supporting Information). The model accurately reproduces the observed onset and decay (solid lines) and thus allows us to directly gauge the exciton lifetime in monolayer CrSBr, for the first time. The best agreement with the experimental data is achieved with an exciton lifetime of 0.5 ps, which is 30 times faster than the decay observed in the bulk. Due to the large oscillator strength of quasi-one-dimensional excitons, we expect an ultrashort radiative lifetime in the monolayer, which we estimate to be of the order of 1 ps (section 5 in the Supporting Information). Yet the decay is unaffected by the pump photon energy, suggesting an important contribution also from nonradiative recombination. As the dynamics are independent of the pump fluence, we can rule out Auger processes, leaving radiative recombination and recombination at defects and surface impurities as the most important decay channels.

Polarization excitation spectroscopy can reveal how the polarizability of the initially photoexcited electron–hole pairs depends on their binding state. To this end, the maximum of Δ*E*_peak_ at *t*_p_ = 0.5 ps (bulk) and *t*_p_ = 0.4 ps (monolayer), respectively, is traced as a function of *h*ν_p_ (Figure 2c,d). For comparison, the data are overlaid with the measured low-temperature photoluminescence spectrum of bulk and monolayer CrSBr as well as the bandgaps obtained from STS (bulk) and calculations (monolayer), respectively. The polarizability of the photoexcited bulk sample (Figure 2c, purple circles) is constant for 1.30 eV ≤ *h*ν_p_ ≤ 1.65 eV, whereas the polarizability dramatically increases for *h*ν_p_ = 1.81 eV, indicating a dominant contribution of electron–hole pairs exhibiting weak or no Coulomb binding. In the monolayer, the polarizability increases upon photoexcitation above the 1s ground state at a photon energy of 1.37 eV. After a plateau at *h*ν_p_ = 1.46 and 1.91 eV, the polarizability decreases by a factor of 3, when *h*ν_p_ is tuned to 2.41 eV. This reduction contrasts with the bulk polarizability spectrum and suggests the observation of a less polarizable, more strongly bound exciton originating from a lower valence band with higher effective masses, which has been theoretically predicted.^22^

While recording Δ*E*_peak_ provides helpful insights into the polarizability and the decay dynamics of photoexcited electron–hole pairs, a quantitative analysis of the binding states of the photoexcited electron–hole pairs calls for complete THz near-field spectroscopy. To this end, the scattered THz waveform is electro-optically sampled for various *h*ν_p_ and *t*_p_. We focus on excitation close to the 1s exciton resonance (*h*ν_p_ = 1.39 eV) and above the bandgap (1.81 eV). Based on the dynamics shown in Figure 2a, the observed ultrafast initial decay of Δ*E*_peak_ for *h*ν_p_ = 1.81 eV has been associated with the dynamics of electron–hole pairs binding into 1s excitons. To test this hypothesis, we compare the pump-induced near-field responses at *t*_p_ = 0.5 and 2.5 ps, which would originate from unbound electron–hole pairs or excitons from energetically lower valence bands and 1s excitons, respectively. Figure 3a depicts the steady-state scattered near-field waveform, *E* (gray), and the pump-induced change, Δ*E* (Φ_p_ = 4 mJ cm^–2^), for *h*ν_p_ = 1.81 eV at *t*_p_ = 0.5 ps (blue) and *h*ν_p_ = 1.39 eV at *t*_p_ = 0.5 ps (red) as well as *h*ν_p_ = 1.81 eV at *t*_p_ = 2.5 ps (dark blue). The first minimum and second maximum of all Δ*E* waveforms are located at zero crossings of the steady-state response. However, the transients clearly differ at positive *t*_EOS_, as highlighted in Figure 3b: both the minimum at *t*_EOS_ = 0.45 ps and the maximum at *t*_EOS_ = 0.70 ps are more strongly pronounced for *h*ν_p_ = 1.81 eV and *t*_p_ = 0.5 ps compared to the other two waveforms, which share similar amplitudes.

**Figure 3:**
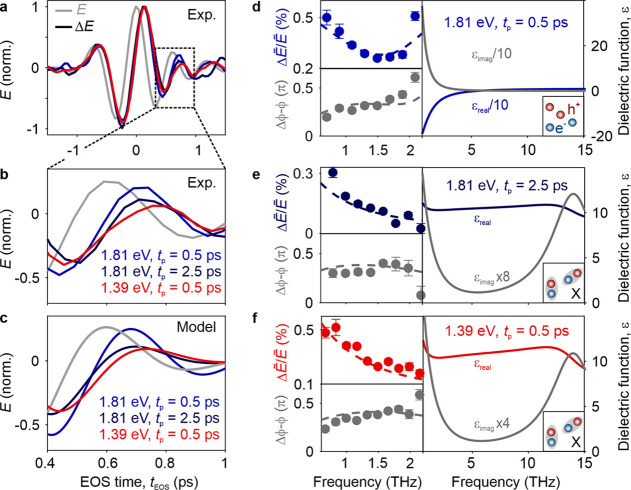
Identifying species of photoexcited electron–hole pairs in bulk CrSBr by THz near-field spectroscopy. (a) Experimental pump-induced changes of the scattered THz waveform, Δ*E*, on bulk CrSBr as a function of the EOS time, *t*_EOS_, for different pump photon energies and pump delay times, *t*_p_. The steady-state near-field response is shown in gray. (b) Zoom-in to the EOS time window between 0.4 and 1 ps. (c) Time-domain near-field responses modeled with the finite-dipole model. (d–f) Relative spectral amplitude, Δ*Ẽ*/*Ẽ* (top left panels), and phase, Δϕ – ϕ (bottom left panels), of the near-field response. The modeled data (dashed lines) calculated with the dielectric functions either comprising a Drude term (d) or two Lorentzians (e, f), shown in the right panels, excellently reproduce the measurements (circles).

This difference is characteristic of distinct changes in the dielectric function. To quantitatively connect the microscopic spectral response with the time-domain signatures observed in Figure 3a, we Fourier transform the waveforms Δ*E* and *E* and consider the corresponding relative spectral amplitude, Δ*Ẽ*/*Ẽ* (Figure 3d–f, top left panels), and phase, Δϕ – ϕ (Figure 3d–f, bottom left panels), in the spectral range of our THz probe pulse. The relative spectral amplitude for *h*ν_p_ = 1.81 eV and *t*_p_ = 0.5 ps features a minimum around 1.6 THz and increases toward the spectral edges, while Δϕ – ϕ (Figure 3d, bottom left) monotonically increases with frequency. This response is markedly different from the two other cases (Figure 3e,f), where Δ*Ẽ*/*Ẽ* decreases monotonically with frequency, while Δϕ – ϕ shows no observable feature and stays around 0.3π.

We analyze how the dielectric response of the sample relates to the observed spectral characteristics by modeling near-field scattering off the photoexcited bulk CrSBr sample with the finite-dipole model (section 6 in the Supporting Information). The terahertz response for *h*ν_p_ = 1.81 eV and *t*_p_ = 0.5 ps can be reproduced best (Figure 3d, dashed line) by a Drude dielectric function shown in Figure 3d (right panel). The modeled spectrum agrees well with the experimental data, indicating the dominant contribution of unbound electron–hole pairs after photoexcitation. In contrast, the Drude response fails to explain the pump-induced change for *h*ν_p_ = 1.81 eV at *t*_p_ = 2.5 ps and *h*ν_p_ = 1.39 eV at *t*_p_ = 0.5 ps (Figure 3e,f, left panels). Therefore, we model the corresponding dielectric function with two Lorentzians, where one oscillator represents the strong, off-resonantly probed 1s–2p transition expected at ∼14 THz. The second, low-energy resonator at 1 THz covers all intraexcitonic transitions from states with large principal quantum numbers (see Figure 3e,f, right panel). The pump-induced response yields excellent agreement with the experiment (Figure 3e,f, left panels). Moreover, in the modeled time-domain data (Figure 3c), the contrast between the Drude and the excitonic near-field response is evident and matches the relative amplitudes of the peaks around *t*_EOS_ = 0.45 and 0.70 ps seen in the experiment (Figure 3b).

In the monolayer sample, effects of finite probing depths and interlayer tunneling are negligible, allowing us to reliably extract the complex nonequilibrium dielectric function in a model-free manner. We record *E* and Δ*E* on a monolayer flake at *t*_p_ = 0.4 ps after resonant excitation of the exciton ground state (*h*ν_p_ = 1.39 eV) with a fluence Φ_p_ = 1.6 mJ cm^–2^ (Figure 4a). The pump-induced change Δ*E* is significantly delayed with respect to *E*. While the first minimum of Δ*E* is enhanced, the second minimum is suppressed. The relative spectral amplitude (Figure 4b, red circles) decreases monotonically, while Δϕ – ϕ (black circles) is mostly flat around 0.3π. From the spectral response of the photoexcited monolayer, we can directly retrieve its complex dielectric function, ε, for the first time, by inverting the finite-dipole model (section 6 in the Supporting Information). The only required assumption is the steady-state dielectric function, ε_eq_, which we found to be constant within our probe spectrum (section 7 in the Supporting Information). For ε_eq_ = 10,^38^ we obtain the nonequilibrium dielectric function depicted in Figure 4c. For frequencies below 1.2 THz, the real part of ε, ε_real_ (teal circles), is increased by ∼30% compared to the steady state (gray dashed line). Above 1.2 THz, ε_real_ is only slightly larger than ε_eq_. The imaginary part, ε_imag_ (purple circles), considerably decreases toward higher frequencies.

**Figure 4:**
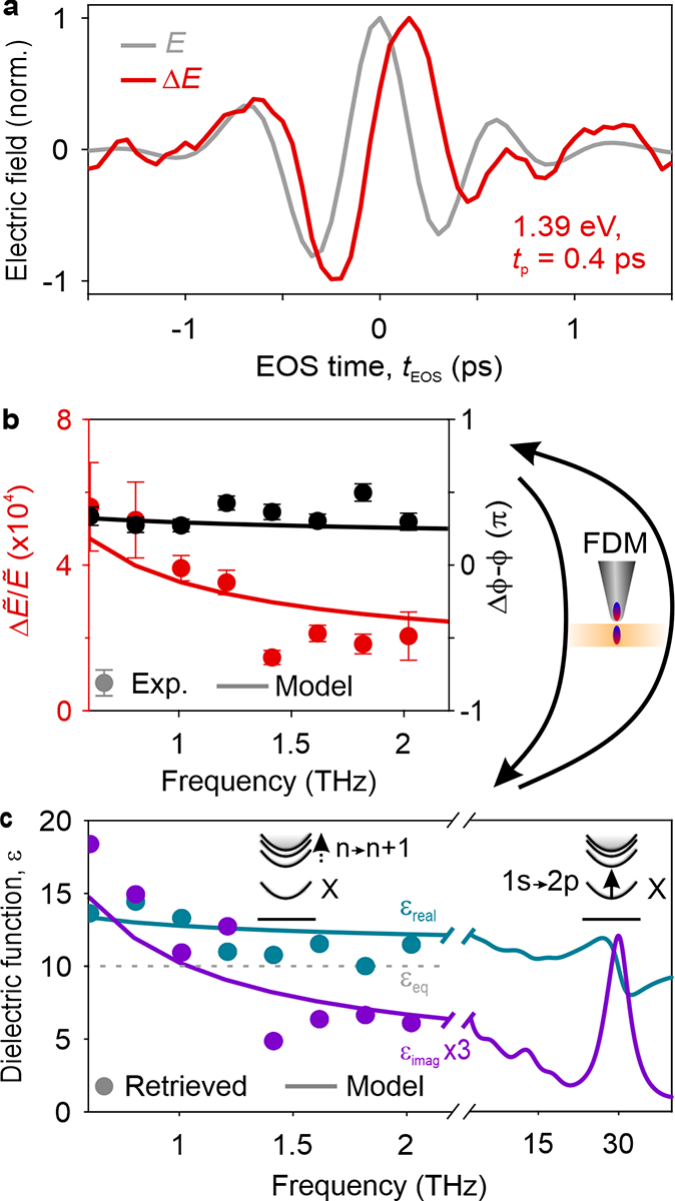
Extracting the complex-valued dielectric function of a photoexcited CrSBr monolayer. (a) Experimental pump-induced change of the scattered THz waveform, Δ*E* (red), for a pump photon energy of 1.39 eV at *t*_p_ = 0.4 ps. The steady-state near-field response, *E*, is shown in gray. (b) Relative spectral amplitude, Δ*Ẽ*/*Ẽ* (red circles), and phase, Δϕ – ϕ (black circles), of the near-field response. (c) Dielectric function obtained for an anisotropic Rytova–Keldysh confinement potential (solid lines) used to calculate the near-field response shown in (b) (solid lines). By numerically inverting the finite-dipole model, we retrieve the complex dielectric function, ε, of the photoexcited monolayer (circles). The assumed equilibrium dielectric function, ε_eq_ = 10, is shown as a gray dashed line.

The increase of ε_real_ and ε_imag_ toward smaller frequencies is reminiscent of a Lorentz oscillator near the low-frequency edge of our probe spectrum, which indicates transitions between highly excited excitons. To corroborate this hypothesis, we model the dielectric function of the nonequilibrium system by calculating the excitonic eigenstates and transition energies for a Rytova–Keldysh potential, considering the anisotropy of the effective mass (section 8 in the Supporting Information). Figure 4c depicts the real (teal line) and imaginary (purple line) parts of the modeled dielectric function. The 1s–2p transition manifests as a peak in ε_imag_ around 27 THz, while the transitions between the more narrowly spaced, higher-energy excitonic states are imprinted in the dielectric function as a steep increase of both ε_real_ and ε_imag_ for decreasing frequency, reliably capturing the shape of the retrieved dielectric function. Lastly, calculating the spectral near-field response of the photoexcited monolayer using the modeled dielectric function (Figure 4b, solid lines) yields excellent agreement with the experimental spectra. These findings provide strong evidence that, at room temperature, the terahertz dielectric response of monolayer CrSBr is dominated by transitions between highly excited, quasi-one-dimensional exciton states.

In conclusion, we explored the ultrafast dynamics of tightly bound electron–hole pairs in the van der Waals magnet CrSBr. In the bulk, we observe an ultrafast relaxation of hot excitons, which may be related to scattering with phonons, defects, or paramagnons and an exciton lifetime of 15 ps. An ultrashort recombination on the time scale of 0.5 ps is revealed in the monolayer, representing the first direct access to the femtosecond dynamics of quasi-one-dimensional excitons in an atomically thin van der Waals magnet. Analyzing the near-field response of bulk CrSBr, we can distinguish the signatures of Coulomb-bound and unbound electron–hole pairs. Furthermore, the nonequilibrium dielectric response of a photoexcited monolayer features the spectral fingerprint of internal transitions between exciton states with high principal quantum numbers. In the future, our near-field spectroscopy approach may be harnessed to investigate the temporal and spectral signatures of coupling of excitons to the various magnetic phases of CrSBr, ultimately even allowing one to image magnetic domains and observe magnetic phase transitions on the nanoscale. Moreover, polarization nanoscopy poses an ideal probe for strain-induced modulations of electronic and magnetic order^39,40^ as well as moiré-twisted van der Waals magnets,^41^ which are promising candidates for next-generation spintronic devices.

## Supporting Information Available

Ultrafast THz near-field spectroscopy setup, tunable optical pump pulses from a noncollinear optical parametric amplifier (NOPA), estimation of the electron–hole pair density, rate-equation model for the ultrafast dynamics of electron–hole pairs, estimating the radiative exciton lifetime in monolayer CrSBr, modeling the spectral near-field response, steady-state nanospectroscopy of monolayer CrSBr, modeling the nonequilibrium dielectric function of monolayer CrSBr, and dependence of the pump–probe dynamics on the pump polarization (PDF)
