# Supplementary material for: Ultrafast Exciton Dynamics in the Atomically Thin van der Waals Magnet CrSBr
Source: Nano Lett. 2024 Mar 20;24(14):4101–7. doi: 10.1021/acs.nanolett.3c05010 (PMC11010225; doi:10.1021/acs.nanolett.3c05010)
Supplement: Supplementary file 1 — nl3c05010_si_001.pdf [file nl3c05010_si_001.pdf]

Supporting information

Ultrafast exciton dynamics in the atomically thin van der Waals magnet CrSBr

Christian Meineke[1], Jakob Schlosser[1], Martin Zizlsperger[1], Marlene Liebich[1], Niloufar Nilforoushan[1], Kseniia Mosina[2], Sophia Terres[3], Alexey Chernikov[3], Zdenek Sofer[2], Markus A. Huber[1], Matthias Florian[4], Mack Kira[4], Florian Dirnberger[3], and Rupert Huber[1]

[1]Department of Physics and Regensburg Center for Ultrafast Nanoscopy (RUN), University of Regensburg, 93040 Regensburg, Germany

[2]Department of Inorganic Chemistry, University of Chemistry and Technology Prague, 166 28 Prague 6, Czech Republic

[3]Institute of Applied Physics and Würzburg-Dresden Cluster of Excellence, Dresden University of Technology, 01187 Dresden, Germany

[4]Department of Electrical Engineering and Computer Science, University of Michigan, Ann Arbor, MI 48109, USA

1. Ultrafast THz near-field spectroscopy setup

The experimental setup is sketched in Figure S1.

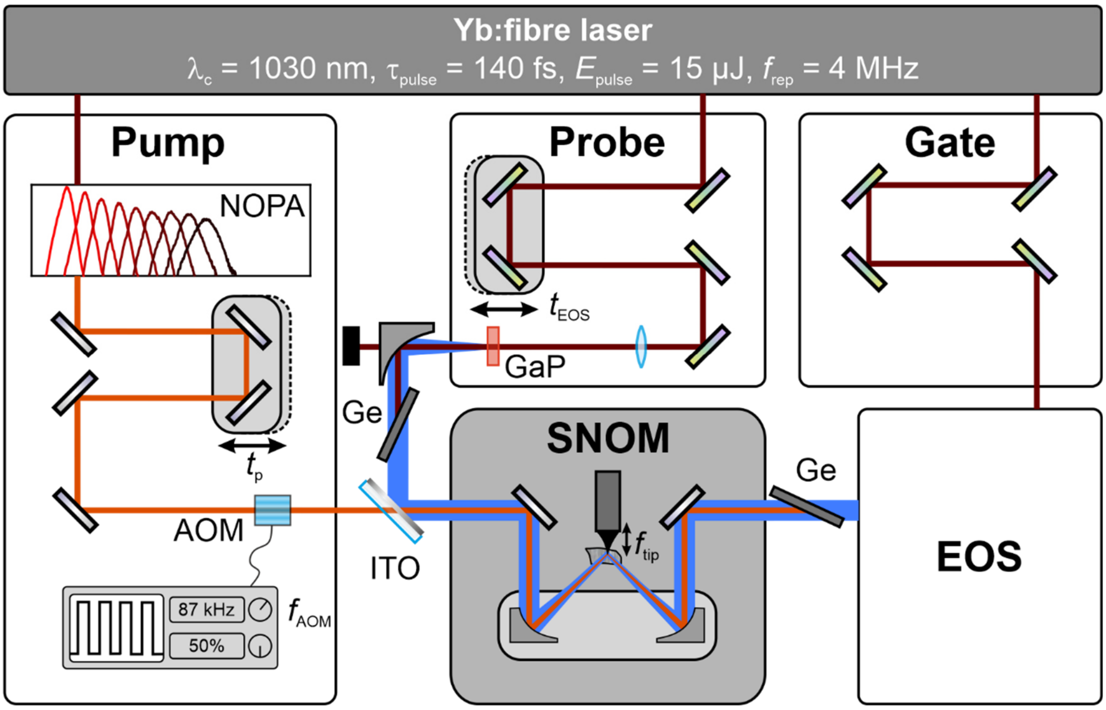

Figure S1. Ultrafast THz near-field spectroscopy setup. A commercial ytterbium fiber amplifier provides near-infrared pulses with a center wavelength of 1030 nm, a pulse duration of 140 fs and a pulse energy of 15 µJ at a repetition rate of 4 MHz (red). We generate phase-locked THz probe pulses (blue) by optical rectification in a gallium phosphide (110) crystal (GaP) with a thickness of 2 mm. Tunable optical pump pulses (orange) are generated in a noncollinear optical parametric amplifier (NOPA, see next section). The THz and optical pulses are superimposed via an indium tin oxide-coated window (ITO) and are focused onto the metallic tip (25PtIr200BH, Rocky Mountain Nanotechnology, LLC) of a commercial near-field microscope (SNOM). The scattered THz radiation containing information on the interaction with the tip-sample junction is collected by a parabolic mirror. The optical pulses are filtered out by a germanium wafer (Ge). The electric field of the probe pulses is detected by electro-optic sampling (EOS) in a GaP crystal (thickness, 2 mm), gated by the laser fundamental (red line). To exclude the far-field background and extract

the pump-induced response, the detected signal is demodulated by a lock-in amplifier either directly at the tapping frequency of the tip, f_{tip}, or, for pump-probe measurements, at the first positive sideband stemming from the pump modulation, $f_{\mathrm{tip}} + f_{\mathrm{AOM}}$. For pump-probe measurements, the pump delay stage was translated (t_{p}) and for spectral analysis of the pump-induced changes of the waveform, the EOS delay was varied (t_{EOS}).

2. Tunable optical pump pulses from a noncollinear optical parametric amplifier (NOPA)

We employ a home-built, two-stage NOPA to generate widely tunable femtosecond pump pulses. A sketch of the setup is shown in Figure S2. The amplified spectra of the pulses that were used to photoexcite the CrSBr samples are shown in Figure S3. We can tune their center photon energy from 1.30 eV to 1.91 eV. For larger photon energies, the generated idler pulses are absorbed in the β-barium borate crystals, hindering effective amplification. The second harmonic of the laser fundamental is centered at 2.4 eV and can also be used to photoexcite the samples.

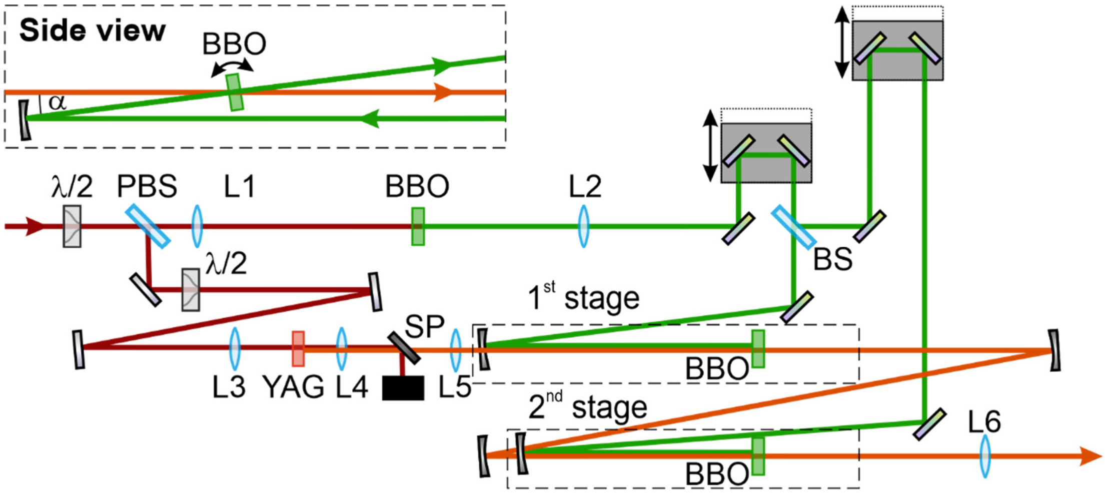

Figure S2. Sketch of the two-stage NOPA. Both stages are seeded by a supercontinuum generated by the laser fundamental in a YAG crystal with a thickness of 5 mm. In another branch, the laser fundamental is frequency-doubled in a β-barium borate crystal (BBO, thickness, 1 mm, angle of cut, 23.4°), that is split by a 50:50 beam splitter (BS) for pumping both amplification stages. In each stage, the pump and seed pulses are noncollinearly focused into β-barium borate crystal with a thickness of 5 mm (angle of cut, 23.4°) to amplify the supercontinuum (side view). λ/2, halfwave plates; PBS, polarizing beam splitter; L1-6, lenses; SP, short pass.

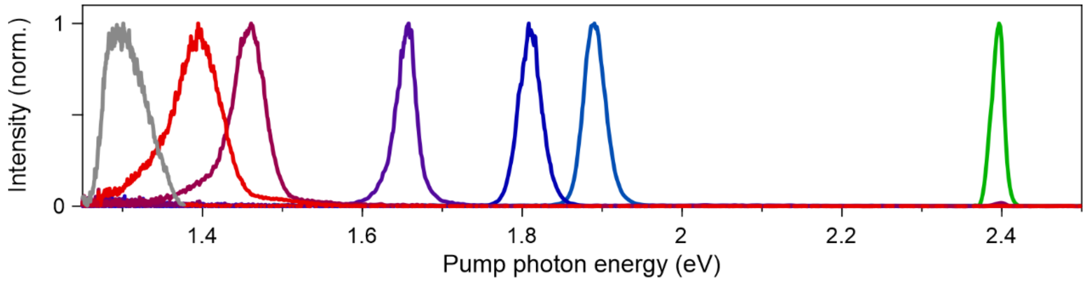

Figure S3. Intensity spectra of the femtosecond pump pulses used in this work. The NOPA allows to generate femtosecond pulses centered from 1.30 eV up to 1.91 eV. The spectrum centered at 2.4 eV is the frequency-doubled laser fundamental, which acts as pump pulse in the NOPA but can also be used to photoexcite the samples.

3. Estimation of the electron-hole pair density

In order to extract the polarizability of one electron-hole pair, the pump-induced near-field response must be normalized to the density of absorbed photons. To this end, we employ the transfer-matrix formalism [41] to estimate the absorption of our Air/CrSBr/SiO_2/p^{++}Si system as a function of photon energy. The dielectric function of CrSBr bulk (thickness, 400 nm) and monolayer (thickness, 1 nm) is taken from calculations using the Bethe-Salpeter equation [22]. The dielectric function of SiO_2 (thickness, 285 nm) is assumed to be constant at 2.11. For the p^{++}Si substrate (boron dopant concentration, $2 \times 10^{19}\text{cm}^{-3} \leq n_\text{B} \leq 1 \times 10^{20}\text{cm}^{-3}$), the refractive index reported in [42] was used. In our near-field microscope, the angle of incidence of the pump pulses with respect to the sample surface is 30°. Furthermore, the pump pulses are polarized perpendicular with respect to the plane of incidence. Figure S4 depicts the calculated absorption spectrum for monolayer and bulk CrSBr (black curves).

In the bulk sample, we consider the finite probing depth by weighting the contribution of the depth-dependent electron-hole pair density, n_eh, with the probing efficiency. Directly after photoexcitation, n_{eh} is given by the Lambert-Beer law, $n_\text{eh}(z) \propto e^{-\alpha z}$. The penetration depth of the pump pulses is given by the dielectric function at the respective pump frequency, ν_p, that is $\alpha^{-1} = \sqrt{\varepsilon}c(2\pi\nu_\text{p}\varepsilon_\text{imag})^{-1}$, where ε_imag denotes the imaginary part of the dielectric function, ε. For the dielectric function given in reference [22] the penetration depth in our pump frequency window covered by our pump pulses ranges between $15\text{ nm} \leq \alpha^{-1} \leq 220\text{ nm}$. As the electron-hole population is probed by an evanescent terahertz field, we assume the probing efficiency to exponentially decay along the growth direction z. A good measure for the decay length is the tapping amplitude of the tip divided by the dielectric constant at the probe frequency [43]. For a dielectric constant equal to 10 [39] and our tapping amplitude of 150 nm, we receive a portion of effectively probed electron-hole pairs,

$$\eta = \frac{\int n_\text{eh}(z)\, e^{-z/(15\text{ nm})}\text{d}z}{\int n_\text{eh}(z)\,\text{d}z} = \frac{\int e^{-\alpha z}\, e^{-z/(15\text{ nm})}\text{d}z}{\int e^{-\alpha z}\,\text{d}z}$$

Figure S4b depicts η as a function of photon energy (red curve) alongside the absorption in bulk CrSBr.

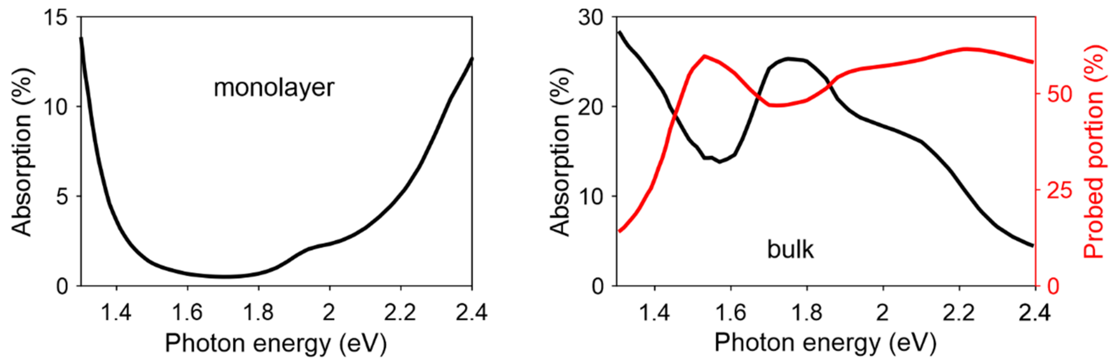

Figure S4. Estimated absorption in bulk and monolayer CrSBr. a) Absorption spectrum of monolayer CrSBr, calculated with the transfer matrix method as described in the text. b) Absorption spectrum of bulk CrSBr (black curve). The portion of effectively probed photoexcited electron-hole pairs for an exponentially decaying probing efficiency is shown as red curve.

4. Rate-equation model for the ultrafast dynamics of electron-hole pairs

In monolayer, CrSBr, we observe an ultrafast, sub-picosecond decay of the pump-induced near-field response. As the timescale of the decay is on a similar order of magnitude as the duration of the pump and gate pulses, we employ a rate-equation model incorporating the finite laser pulse durations to demonstrate that the rapid decay of ΔE_{peak} lies well within our temporal resolution. We simulate the femtosecond dynamics of electron-hole pairs in monolayer CrSBr with a straightforward rate equation for the density of electron-hole pairs $n_{\text{eh}}(t)$:

$$\frac{dn_{\text{eh}}(t)}{dt} = S_{\text{pump}}(t) - \frac{n_{\text{eh}}(t)}{\tau}.$$

Here, S_{pump} is a source term with the shape of the temporal intensity envelope of the pump pulse, modeled as a Gaussian with a full width at half maximum (FWHM) of 100 fs. The photoexcited population decays with the lifetime τ, representing the only fit parameter in this model. We then assume, that ΔE_{peak} is directly proportional to the density $n_{\text{eh}}(t)$, which is justified, when the polarizability of the electron-hole pairs does not change considerably. In order to account for the finite detection bandwidth, the resulting dynamics are convoluted with the intensity envelope of the EOS gate pulses, which exhibit a FWHM duration of 140 fs. As shown in the main text, in this way, we can reliably reproduce both the onset and the decay of ΔE_{peak}.

5. Estimating the radiative exciton lifetime in monolayer CrSBr

We estimate the intrinsic radiative lifetime τ_{rad}^{0} of excitons in monolayer CrSBr with a simplistic two-band model in two dimensions following reference [43]:

$$\tau_{\mathrm{rad}}^{0} = \frac{4\pi\varepsilon_0\varepsilon\hbar}{2k_0}\left(\frac{E_{\mathrm{X}}}{e\hbar v}\right)^2 (a_{\mathrm{B}}^{\mathrm{2D}})^2.$$

Here, $k_0 = E_{\mathrm{X}}\sqrt{\varepsilon}/(\hbar c)$ is the wave-vector of the emitted light, E_{X} the exciton transition energy, c the speed of light, e the electron charge, $v = \sqrt{E_{\mathrm{g}}/(2m^*)}$ the Kane velocity and $a_{\mathrm{B}}^{\mathrm{2D}}$ the two-dimensional Bohr radius. We account for the anisotropic nature of the exciton by taking into account the anisotropic effective masses along the a and b crystallographic axes, which result in different Bohr radii r_a and r_b, respectively. We then modify the effective Bohr radius $a_{\mathrm{B}}^{\mathrm{2D}}$ considering the exciton as an ellipse with principal radii r_a and r_b, such that

$$a_{\mathrm{B}}^{\mathrm{2D}} = \sqrt{r_a r_b}.$$

Analogously, we calculate the average effective mass, $m^* = \sqrt{m_a m_b}$, where m_a and m_b are the effective electron masses along the a and b axis, respectively. For the effective masses given in reference [22], $\varepsilon = 10$ [39], $E_{\mathrm{X}} = 1.37$ eV and $E_{\mathrm{g}} = 2.1$ eV [22] we obtain a radiative lifetime of $\tau_{\mathrm{rad}}^{0} \approx 0.8$ ps.

6. Modeling the spectral near-field response

In order to model the near-field interaction in Figure 3 and Figure 4, we make use of the finite-dipole model [44,45], simulating the frequency-dependent complex scattering amplitude E, as well as the pump-induced change $\Delta E = E_{\text{pumped}} - E_{\text{unpumped}}$. As input parameters, we determine the tapping amplitude (150 nm), the radius of curvature of the tip (20 nm), the tip spheroid length $2L$ (720 nm), and an empirical geometry factor g ($0.7e^{0.06i}$), as described in previous studies using the finite-dipole model in the THz frequency range [46,47]. Finally, our sample is modeled as a layered system, where the CrSBr sample located on SiO_2 is embedded in infinite half-spaces of air.

The bulk sample is modeled with a thickness of 400 nm and a dielectric function, which is either parametrized as a Drude response,

$$\varepsilon(\omega) = \varepsilon_{\text{eq}} - \frac{\omega_p^2}{\omega^2 + i\frac{\omega}{\tau_{\text{sc}}}}$$

with a constant equilibrium dielectric function $\varepsilon_{\text{eq}} = 10$, plasma frequency $\omega_{\text{p}} = 2\pi\nu_{\text{p}} = \sqrt{\frac{ne^2}{m^*\varepsilon_0}}$, Drude scattering time τ_{sc}, charge-carrier density n, elementary charge e, effective mass m^*, and vacuum permittivity ε_0, or two Lorentzian oscillators,

$$\varepsilon(\omega) = \varepsilon_{\text{eq}} + \sum_k \frac{f_k \omega_{\text{res},k}^2}{\omega_{\text{res},k}^2 - \omega^2 - i\beta_k\omega}$$

with resonant angular frequency $\omega_{\text{res},k}^2$, damping coefficient β_k, and oscillator strength f_k, for each oscillator k. The parameters used to reproduce the pump-induced near-field response of bulk CrSBr are listed in Table T1. The theoretical relative amplitude spectrum obtained by the Drude response is rescaled by a factor of 0.11 to obtain optimal agreement with the experimental data.

To directly extract the complex dielectric function of a CrSBr monolayer (thickness, 1 nm), we inverted the finite-dipole model with the algorithm described in [44]. To guarantee an unambiguous result, we allow for imaginary parts, $\varepsilon_{\text{imag}} > 0$, while the real part, $\varepsilon_{\text{real}}$ may assume any real number.

**Drude**	**hν_p = 1.81 eV, t_p = 0.5 ps**	**Lorentz**	**hν_p = 1.81 eV, t_p = 2.5 ps**	**hν_p = 1.39 eV, t_p = 0.5 ps**
ν_p (THz)	18	$\nu_{res,1}$ (THz)	1	1
τ_{sc} (fs)	85	f_1	20	35
		β_1 (THz)	20	20
		$\nu_{res,2}$ (THz)	14	14
		f_2	150	350
		β_2 (THz)	30	30

Table T1. Parameters of the dielectric functions used to reproduce the spectral near-field responses in bulk CrSBr.

7. Steady-state nano-spectroscopy of monolayer CrSBr

In order to extract the nonequilibrium dielectric function of a photoexcited monolayer of CrSBr, it is helpful to first investigate the steady-state response. Here, we perform nano-spectroscopy by recording the scattered THz waveform on a monolayer without optical excitation. When the spectral response demodulated at the n-th harmonic of the tip oscillation frequency is divided by the response at the (n-1)-th harmonic, one can identify polar oscillators in the sample and, in this way, obtain information about the shape of the equilibrium dielectric function, as shown in reference [49].

Figure S5a depicts the electro-optically detected scattered THz field demodulated at the second and third harmonic of the tip oscillation frequency, E_2 and E_3, recoded on an unpumped monolayer of CrSBr. E_2 and E_3 oscillate perfectly in phase, while the amplitude of E_2 exceeds the one of E_3. This becomes particularly clear in Figure S5b, where the relative spectral amplitude, $\tilde{E}_3/\tilde{E}_2$, and phase, $\phi_3 - \phi_2$, are shown. Both relative amplitude and phase are flat, corroborating that there are no spectral features in the equilibrium dielectric function. Thus, the latter can be assumed to be flat.

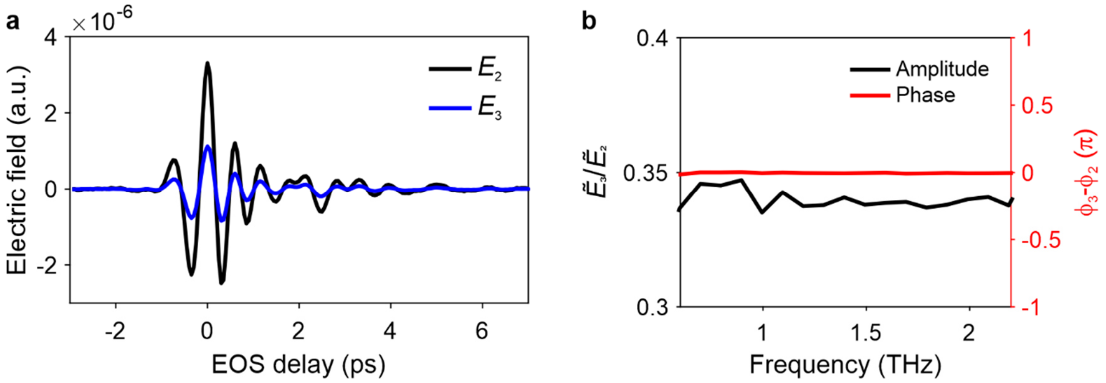

Figure S5. Steady-state nano-spectroscopy of monolayer CrSBr. a) Electro-optically detected scattered THz near field at the second and third harmonic of the tip oscillation frequency, E_2 and E_3. b) Relative spectral amplitude, $\tilde{E}_3/\tilde{E}_2$, and phase, $\phi_3 - \phi_2$.

8. Modeling the nonequilibrium dielectric function of monolayer CrSBr

To model the dielectric response in the monolayer, we solve the two-dimensional Schrödinger equation using the finite difference method implemented in an open-source code available under [https://github.com/LaurentNevou/Q_Schrodinger2D_demo]. The strongly screened potential is approximated by an anisotropic Rytova-Keldysh potential [50,51] of the form

$$V = -\frac{1}{4\pi\epsilon_0}\frac{e^2}{r_0}\frac{\pi}{2}\left[H_0\left(\frac{\kappa r}{r_0}\right) - Y_0\left(\frac{\kappa r}{r_0}\right)\right].$$

Here, H_0 and Y_0 are the zero-order Struve and Neumann special functions, $\kappa = (\varepsilon_{air} + \varepsilon_{SiO_2})/2$ represents the screening of the air and SiO_2 hemispheres the monolayer is embedded in and $r_0 = 2\pi \times 1$ nm is a measure of the dielectric screening length in the CrSBr monolayer, taken from reference [52]. To account for the strong anisotropy of the reduced effective mass μ, we perform a transformation of coordinates analogous to references [50,53],

$$r \rightarrow r\sqrt{1 + \beta\, cos\theta},$$

where $\beta = (\mu_a - \mu_b)/(\mu_a + \mu_b)$ and θ is the polar angle. The respective reduced effective masses along the crystallographic a and b axis, μ_a and μ_b are taken from reference [22]. Note, that this approximation neglects the anisotropy of the dielectric constant, which is justified by the much stronger anisotropy of the effective mass.

The pump-induced change of the dielectric function was then calculated as follows: The transition dipole matrix elements of the obtained eigenstates multiplied with the thermal population at room temperature are used as measure of the oscillator strength of Lorentz lines centered at the respective transition energies. A width of 20 meV was assumed for every line. The obtained pump-induced change of the dielectric function was used in the finite-dipole model and multiplied by a constant real factor to match the experimental data.

9. Dependence of the pump-probe dynamics on the pump polarization

We investigated the dependence of the decay of the pump-probe signal on the polarization of the pump pulses. To this end, we rotated the bulk CrSBr sample by 90° with respect to the position used for the measurements presented in the main text, resulting in a pump polarization aligned with the crystallographic a-axis. In Figure S5, we compare the dynamics of ΔE_{peak} for pump photon energies of $h\nu_{\mathrm{p}} = 1.39$ eV (red) and $h\nu_{\mathrm{p}} = 1.81$ eV (blue) for a pump polarization along the a-axis (dashed lines) and b-axis (solid lines). The data is divided by the respective pump fluence. As expected, owing to the weak absorption the strength of the pump-induced signal is strongly suppressed for excitation along the a-axis. However, the dynamics are the same for a- and b-axis.

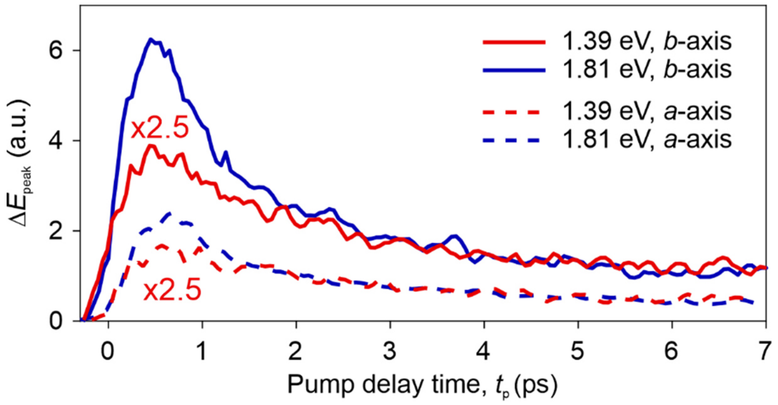

Figure S5. Decay dynamics in bulk CrSBr for photoexcitation along different crystal axes. Pump-induced change of the near-field response of bulk CrSBr recorded at $t_{\mathrm{EOS}} = 0.15$ ps, ΔE_{peak}, as a function of pump delay time, t_{p}, for excitation photon energies of 1.39 eV (red) 1.81 eV (blue). The polarization dynamics for photoexcitation polarized along the crystallographic a-axis (dashed lines) and b-axis (solid lines) are compared. The data is normalized to the respective pump fluence.

REFERENCES

41. Zhan, T., Shi, X., Dai, Y., Liu, X. & Zi, J. Transfer matrix method for optics in graphene layers. *J. Phys. Condens. Matter* **25**, (2013).
42. Schinke, C. *et al*. Uncertainty analysis for the coefficient of band-to-band absorption of crystalline silicon. *AIP Advances* **5**, 67168 (2015).
43. Robert, C. *et al.* Exciton radiative lifetime in transition metal dichalcogenide monolayers. *Phys. Rev. B* **93**, 205423 (2016).
44. Mooshammer, F. *et al.* Nanoscale Near-Field Tomography of Surface States on $(Bi_{0.5}Sb_{0.5})_2Te_3$. *Nano Lett.* **18**, 7515–7523 (2018).
45. Cvitkovic, A., Ocelic, N. & Hillenbrand, R. Analytical model for quantitative prediction of material contrasts in scattering-type near-field optical microscopy. *Opt. Express* **15**, 8550 (2007).
46. Hauer, B., Engelhardt, A. P. & Taubner, T. Quasi-analytical model for scattering infrared nearfield microscopy on layered systems. *Opt. Express* **20**, 13173 (2012).
47. Liewald, C. *et al.* All-electronic terahertz nanoscopy. *Optica* **5**, 159–163 (2018).
48. Aghamiri, N. A. *et al.* Hyperspectral time-domain terahertz nano-imaging. *Opt. Express* **27**, 24231 (2019).
49. Mester, L., Govyadinov, A. A. & Hillenbrand, R. High-fidelity nano-FTIR spectroscopy by on-pixel normalization of signal harmonics. *Nanophotonics* **11**, 377–390 (2022).
50. Rodin, A. S., Carvalho, A. & Castro Neto, A. H. Excitons in anisotropic two-dimensional semiconducting crystals. *Phys. Rev. B* **90**, 075429 (2014).
51. Tuan, D. Van, Yang, M. & Dery, H. Coulomb interaction in monolayer transition-metal dichalcogenides. *Phys. Rev. B* **98**, 125308 (2018).

52. Kamban, H. C., Pedersen, T. G. & Peres, N. M. R. Anisotropic Stark shift, field-induced dissociation, and electroabsorption of excitons in phosphorene. *Phys. Rev. B* **102**, 115305 (2020).

53. Qian, T.-X., Zhou, J., Cai, T.-Y. & Ju, S. Anisotropic electron-hole excitation and large linear dichroism in the two-dimensional ferromagnet CrSBr with in-plane magnetization. *Phys. Rev. Res.* **5**, 033143 (2023).
